# High-normal liver enzyme levels in early pregnancy predispose the risk of gestational hypertension and preeclampsia: A prospective cohort study

**DOI:** 10.3389/fcvm.2022.963957

**Published:** 2022-09-12

**Authors:** Yi Zhang, Chen Sheng, Dingmei Wang, Xiaotian Chen, Yuan Jiang, Yalan Dou, Yin Wang, Mengru Li, Hongyan Chen, Wennan He, Weili Yan, Guoying Huang

**Affiliations:** ^1^Department of Clinical Epidemiology and Clinical Trial Unit, Children's Hospital of Fudan University, National Children's Medical Center, Shanghai, China; ^2^Pediatric Heart Center, Children's Hospital of Fudan University, National Children's Medical Center, Shanghai, China; ^3^Shanghai Key Laboratory of Birth Defects, Shanghai, China; ^4^Research Unit of Early Intervention of Genetically Related Childhood Cardiovascular Diseases (2018RU002), Chinese Academy of Medical Sciences, Shanghai, China

**Keywords:** liver enzymes, early pregnancy, gestational hypertension, prospective cohort study, preeclampsia

## Abstract

**Background:**

Gestational hypertension (GH) and preeclampsia (PE) are severe adverse gestational complications. Previous studies supported potential link between elevated liver enzyme levels and GH and PE. However, given the transient physiological reduction of liver enzyme levels in pregnancy, little is known whether the associations of the high-normal liver enzyme levels in early pregnancy with GH and PE exist in pregnant women.

**Methods:**

Pregnant women in this study came from a sub-cohort of Shanghai Preconception Cohort, who were with four liver enzyme levels examined at 9–13 gestational weeks and without established liver diseases, hypertension and preeclampsia. After exclusion of pregnant women with clinically-abnormal liver enzyme levels in the current pregnancy, associations of liver enzyme levels, including alkaline phosphatase (ALP), alanine transaminase (ALT), aspartate aminotransferase (AST) and gamma-glutamyl transpeptidase (GGT), with GH and PE status were assessed by multivariable log-binomial regression. Population attributable fraction was measured to estimate the fractions of GH and PE that were attributable to the high-normal liver enzyme levels.

**Results:**

Among 5,685 pregnant women 160 (2.8%) and 244 (4.3%) developed GH and PE, respectively. After adjustment for potential covariates, higher ALP, ALT and GGT levels were significantly associated with the risk of GH (adjusted risk ratio (aRR):1.21 [95% confidence interval, 1.05–1.38]; 1.21 [1.05–1.38]; and 1.23 [1.09–1.39]), as well as the risk of PE(1.21 [1.13–1.29]; 1.15 [1.03–1.28]; 1.28 [1.16–1.41]), respectively. The cumulative population attributable fraction of carrying one or more high-normal liver enzyme levels (at 80th percentile or over) was 31.4% for GH and 23.2% for PE, respectively.

**Conclusion:**

Higher ALT, ALP and GGT levels within the normal range in early pregnancy are associated with increased risk of GH and PE. The documented associations provide new insight to the role of hepatobiliary function in GH and PE pathogenesis.

## Introduction

Hypertensive disorders of pregnancy (HDP) includes preeclampsia (PE) and gestational hypertension (GH) diagnosed over the 20th week of gestation. PE is a severe clinical syndrome affecting 5–7% of pregnant women, which remains responsible for over 70,000 maternal deaths and 500,000 fetal deaths worldwide every year (1). There is few effective clinical management for GH and PE except for close monitoring of maternal symptoms (2). Therefore, investigating modifiable risk factors remains valuable for early prevention of HDP.

Abnormal liver function in pregnancy has been recognized as an important risk factor for GH and PE (2, 3). Liver enzymes, including aspartate aminotransferase (AST), alanine aminotransferase (ALT), alkaline phosphatase (ALP) and γ-glutamyl transferase (GGT), are considered as biomarkers of hepatocellular damage and cholestatic liver disease (4). Recent multi-center cohort studies have shown that abnormal levels of ALT and AST in pregnancy conferred 1.12- to 3-fold increase of PE risk (5–7). A prospective cohort study has demonstrated that abnormally elevated ALP and GGT levels are positively associated with blood pressure pattern during pregnancy (8). According to International Society for the Study of Hypertension in Pregnancy (ISSHP), pregnant women with abnormal ALT and AST levels (any one of which > 40 U/L) warrant specific attention and antenatal care for their high risk of HDP (9).

The establishment of clinical liver enzyme cutoffs for defining abnormal status were mainly based on the non-pregnant population, however, the liver enzymes physiologically decrease during pregnancy (10). In pregnant women, AST, ALT, and GGT levels were about 20% lower compared with the currently used clinical reference ranges for general population (11), indicating that liver function tests of pregnant women whose liver enzyme levels lying among the top 20 percentiles might be misclassified as normal according to the current references. Despite this, it remains unclear whether the liver enzyme levels in the clinically normal range are associated with GH and PE among pregnant women. With the advantage of our prospective cohort, we aim to evaluate the associations of liver enzyme levels in normal range with the subsequent risk of GH and PE.

## Materials and methods

### Study population

Our study sample was from one study center of ongoing Shanghai Preconception Cohort (SPCC; NCT02737644) (12), at which liver enzyme levels are routinely examined in early pregnancy (between 9 and 13 gestational weeks) at the first prenatal care. After excluding of participants who met any of the following three criteria, the pregnant women with clinically normal liver functions were included for the current study: ① history of hypertension or PE; ②pregnant women with severe liver diseases (hepatic viral infections, intrahepatic cholestasis, pregnancy complicated with liver damage, polycystic liver disease, pregnancy complicated with liver failure and liver cancer); ③ hepatobiliary abnormality defined as any one of the liver enzyme levels exceeding the American Board of Internal Medicine Laboratory Test Reference Ranges (13) (ALT > 40 U/L, AST > 40 U/L, ALP > 120 U/L, and GGT > 40 U/L). The study protocol was approved by the institutional Ethics Committee (No. 201649). Participants in this study had no involvement in the design of current study.

### Diagnosis of GH and PE

Routine blood pressure and proteinuria measurements were derived from antenatal medical records and GH and PE were diagnosed according to the criteria of the International Society for the Study of Hypertension in Pregnancy (ISSHP) (14).

### Exposures and covariates

Liver enzymes including ALP, ALT, AST and GGT were extracted from the hospital medical record system that had been routinely examined at the first antenatal care visit at the hospital. As described elsewhere (12), information about baseline characteristics of participants of this study was obtained by a standardized self-administered questionnaire at recruitment at the first antenatal visit. Maternal body mass index (BMI) before pregnancy was calculated by self-report pre-pregnancy body weight and height and categorized as normal (<24 kg/m^2^) or overweight/obese (≥ 24 kg/m^2^) according to the definition of the Chinese population (15). The gestational week at recruitment was assessed by the date of last menstrual period in combination with routine ultrasound examination at the first trimester of antenatal care. Smoking exposure was defined as self-smoker or exposure to secondhand cigarette smoking. Alcohol drinking was defined as drinking of any alcoholic beverages within 3 months before pregnancy or during pregnancy. Folic acid supplementation was defined as regular intake of folic acid tablets or multivitamins containing folic acid in early pregnancy.

### Statistical analysis

Continuous variables with normal distribution were presented as mean (standard deviation [SD]), while variables with skewed distribution were described as median and interqurtile ranges (IQR). Categorical variables were presented as absolute numbers and percentages (%). All baseline characteristics of pregnant women with GH and PE and non-HDP were compared by Student's *t*-tests and Mann–Whitney U rank-sum tests for continuous variables and Chi-square tests for categorical ones.

The associations of liver enzyme levels (continuous variables, per SD increment) with GH and PE risk were evaluated by multivariable log-binomial regression. Considering risk factors of GH and PE reported in previous studies, our study adjusted age (years), preconception-BMI (normal: BMI <24 kg/m^2^ or overweight/obese: BMI ≥ 24 kg/m^2^), supplementation of folic acid before pregnancy (yes/no), smoking exposure during gestation (yes/no), whether gravidity > 1 (yes/no), gestational week at the 1st antenatal visit and fasting glucose at the 1st antenatal visit as covariates. The continuous liver enzyme levels were further categorized into quintiles and were analyzed as dummy variables in reference to the 1st quintile levels to indicate the risk of outcomes for each category. The 5th quintile enzyme levels, at 80th percentiles or obove was defined as high-normal levels. Unadjusted risk ratio (RR) and adjusted risk ratio (aRR) and 95% confidence interval (95% CI) were reported. Then, we used restricted cubic spline (RCS) models with three knots placed at the 10, 50, and 90th percentiles to further investigate the nonlinearity of the associations of continuous liver enzymes with the GH and PE. To quantify the attributable risk of GH and PE according to carrying one to four high-normal liver enzymes, population attributable fraction (PAF) was estimated using packages “punafcc” (16) based on the previous multivariable log-binomial models. Adjusted RRs, PAF and 95% CIs were reported for exposed one to four high-normal liver enzymes levels (ALP > 62 U/L, ALT > 25 U/L, AST > 22 U/L, and GGT > 20 U/L) in reference to those with four liver enzymes below 5th quintiles (80th pertentiles).

To verify the robustness of the associations between liver enzymes and GH and PE, sensitivity analyses were conducted by including participants with established liver diseases and clinically-abnormal liver enzyme levels. Characteristics of participants included in the main analysis were compared with those excluded to evaluate the potential selection bias. All data were analyzed using the Stata version 16.0 (Stata Corp LLC, USA).

## Results

### Participant characteristics

**Figure 1:**
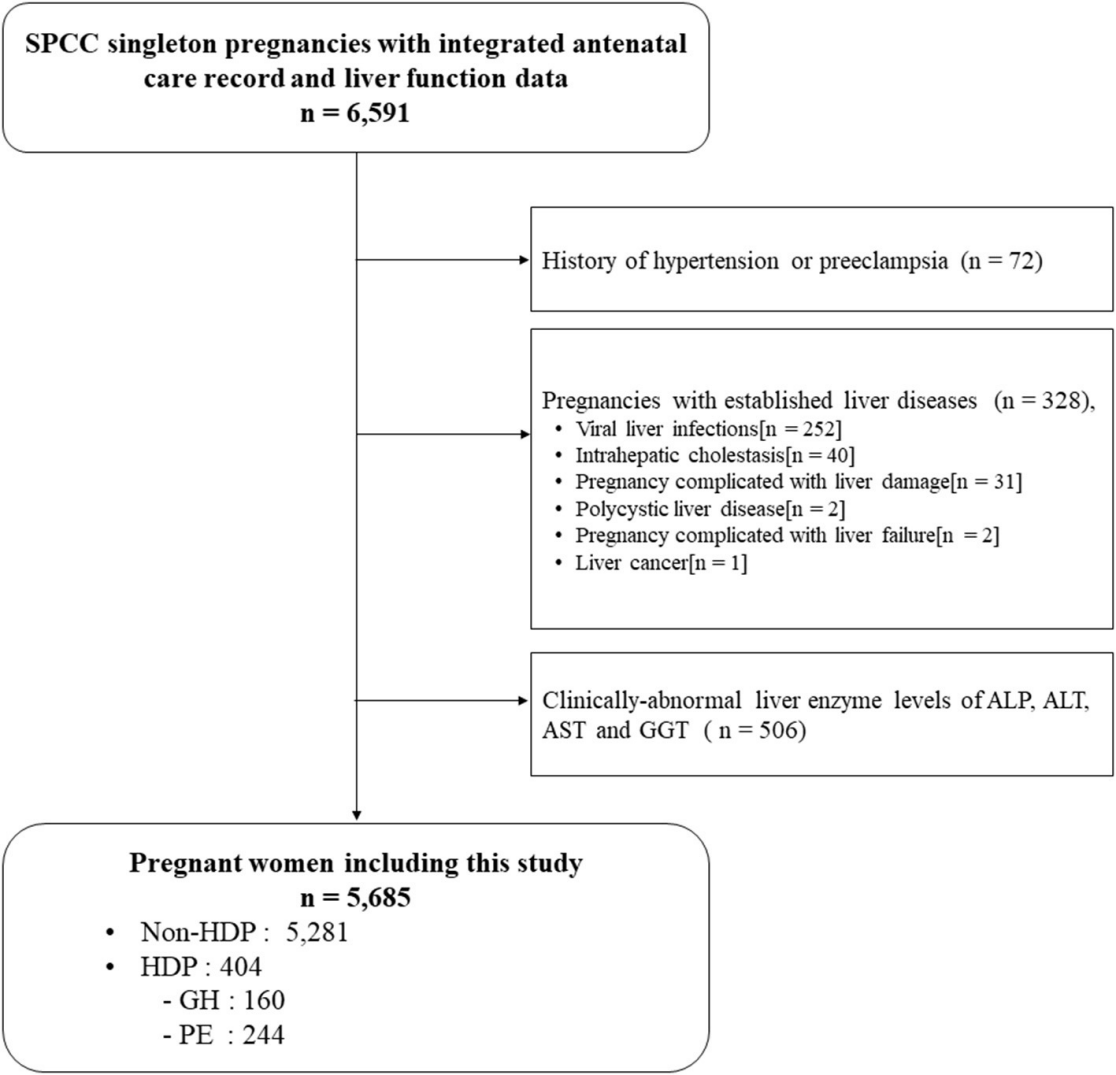
Flow chart of participants. HDP, hypertensive disorders of pregnancy; GH, Gestational hypertension; PE, Preeclampsia.

A total of 6,591 participants had complete GH and PE diagnosis record and liver enzyme data, of which 400 participants were excluded because of hypertension and PE history (n = 72), and established liver diseases during this pregnancy (*n* = 328). Further, 506 participants with clinically-abnormal liver enzyme levels were excluded. Finally, 5,685 pregnant women were included for analysis, among which 160 (2.8%) developed GH and 244 (4.3%) developed PE, respectively (Figure 1).

Table 1 summarized the characteristics of the overall study sample and the group of GH and PE, respectively. For all subjects, the median gestational week at the first clinical antenatal care was 11 weeks and the average age was 31.0 years. Compared with non-HDP group, pregnant women with GH and PE showed higher levels of body mass index, fasting glucose, ALP, ALT and GGT (all *P* ≤ 0.005) at the first antenatal care, were more likely to be overweight/obese before pregnancy, and develop GDM. The four liver enzyme levels in early pregnancy among non-HDP, GH and PE showed approximately normal distribution with slightly skewed to the right (Supplementary Figure S1).

**Table 1:** Demographic characteristics of the study population.

**Characteristics**	**Overall** ***n* = 5,685**	**Non-HDP** ***n* = 5,281**	**GH** ***n* = 160**	***P*_GH vs. non−HDP_**	**PE** ***n* = 244**	***P*_PE vs. non−HDP_**
Age (years)^*^	31.0 (4.0)	30.9 (4.0)	31.4 (4.4)	0.12	31.9 (4.5)	<0.001
Gestational week (weeks)^†^	11.0 (9.6, 12.4)	11.0 (9.6, 12.4)	11.0 (9.4, 12.1)	0.35	11.0 (9.6, 12.3)	0.63
Gravidity >1 [n, (%)]	2,563 (45.1)	2,403 (45.5)	62 (38.8)	0.11	98 (40.2)	0.13
Pre-conceptional BMI (kg/m^2^)^*^	21.1 (2.8)	20.9 (2.7)	23.3 (3.8)	<0.001	22.5 (3.3)	<0.001
Overweight or Obesity before pregnancy [n, (%)]	797 (14.0)	668 (12.6)	58 (36.2)	<0.001	71 (29.1)	<0.001
BMI in the 1st antenatal care (kg/m^2^)^*^	21.6 (2.9)	21.4 (2.8)	23.9 (3.8)	<0.001	23.1 (3.5)	<0.001
History of preconception folic acid intake [n, (%)]	1,246 (21.9)	1,167 (22.1)	29 (18.1)	0.25	50 (20.5)	0.64
Folic acid supplement during early gestation [n, (%)]	5,275 (92.8)	4,903 (92.8)	144 (90.0)	0.16	228 (93.4)	0.80
Exposure to cigarette in early gestation [n, (%)]	623 (11.2)	583 (11.1)	14 (8.8)	0.44	35 (14.4)	0.12
History of alcohol use in early gestation [n, (%)]	477 (8.4)	453 (8.6)	9 (5.6)	0.25	15 (6.1)	0.24
Diagnosed gestational diabetes mellitus [n, (%)]	733 (12.9)	652 (12.3)	36 (22.5)	<0.001	45 (18.4)	0.011
Serum markers at the 1st antenatal visit						
Glucose, mean (mmol/L)^*^	4.5 (0.4)	4.5 (0.4)	4.7 (0.5)	<0.001	4.6 (0.5)	0.026
ALP, mean (U/L)^*^	51.2 (11.3)	51.0 (11.2)	54.6 (12.4)	<0.001	54.1 (12.0)	<0.001
ALT, mean (U/L)^*^	15.0 (7.0)	14.9 (7.0)	17.2 (7.5)	<0.001	16.5 (7.7)	<0.001
AST, mean (U/L)^*^	17.3 (4.0)	17.2 (4.0)	17.8 (4.3)	0.090	17.7 (4.4)	0.10
GGT, mean (U/L)^*^	14.1 (5.9)	13.9 (5.8)	16.4 (7.1)	<0.001	16.6 (7.5)	<0.001

GH, gestational hypertension; PE, preeclampsia; HDP, hypertensive disorders of pregnancy; BMI, body mass index; ALT, alanine aminotransferase; AST, aspartate aminotransferase; GGT, γ-glutamyl transferase; ALP, alkaline phosphatase.

* Continuous variables with normally distributed are presented as means ± standard deviation, and the differences are tested by the Student's t-tests.

† Skewed continuous variables are presented as medians and IQRs, and the differences are tested by the Mann-Whitney U-tests.

### Associations of maternal liver enzyme levels with GH and PE

**Figure 2:**
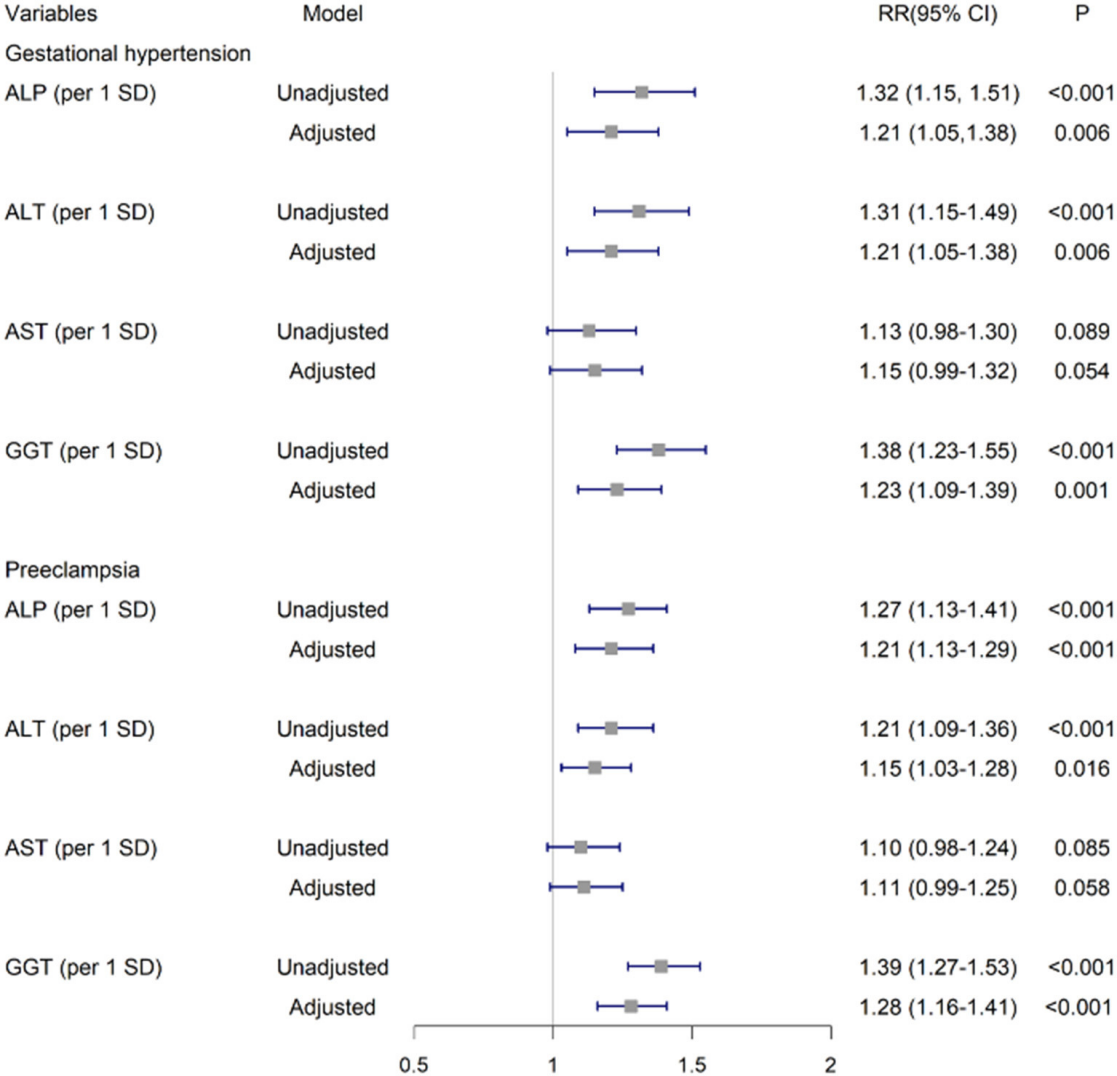
Associations of ALP, ALT, AST and GGT in early pregnancy with GH and PE. The risk ratios and 95% CIs were estimated by multivariable log-binomial regression adjusting for age (years), preconception-BMI (normal: BMI <24 kg/m^2^ or overweight/obese: BMI ≥ 24 kg/m^2^), supplementation of folic acid before pregnancy (yes/no), smoking exposure during gestation (yes/no), whether gravidity>1 (yes/no), gestational week and fasting glucose at the 1^st^ antenatal visit. GH, Gestational hypertension; PE, Preeclampsia; GGT, γ-glutamyl transferase; ALT, alanine aminotransferase; ALP, alkaline phosphatase; AST, aspartate aminotransferase.

After adjustment for covariates, per SD of ALP, ALT and GGT were positively associated with GH as (aRR, 1.21 [95%CI, 1.05–1.38]; 1.21 [1.05–1.38]; and 1.23 [1.09–1.39], respectively). These significant associations persisted for PE (1.21 [1.13–1.29], 1.15 [1.03–1.28],and 1.28 [1.16–1.41], respectively). No significant associations were found for AST as continuous variables with GH or PE (Figure 2).

Table 2 showed adjusted risk ratios and 95% confidence intervals for associations of quintiles of liver enzyme levels in early pregnancy with GH and PE. In reference to the 1st quintile, the 5th quintile of ALP, ALT and GGT levels were significantly associated with GH aRR 1.91 [95% CI, 1.16–3.14], 2.10 [1.25–3.46], and 1.96 [1.28–3.02], respectively. Similar positive associations were observed for quintiled ALP and GGT with PE, respectively (1.93 [1.27–2.91] and 2.31 [1.59–3.35]). Of note, the 5th quintile levels of these liver enzymes were lower than the current clinical cutoffs for abnormal liver function (13) and previously suggested reference ranges for early pregnant women (10) (Supplementary Table S1).

**Table 2:** Associations of quintiled liver enzyme levels in early pregnancy with risk of GH and PE.

Variables^*^	**GH**			**PE**		
	**N(%)**	**Unadjusted** ** RR (95% CI)**	**aRR^†^ (95% CI)**	**N(%)**	**Unadjusted** ** RR (95% CI)**	**aRR^†^ 95% CI**
**ALP**						
Q1 (0–43.0 U/L)	23/1,186(1.9)	1.00	1.00	34/1,197(2.8)	1.00	1.00
Q2 (43.1–48.9 U/L)	20/1,018(1.9)	1.01 (0.56–1.83)	1.01 (0.55–1.82)	43/1,041(4.1)	1.45 (0.93–2.26)	1.37 (0.88–2.14)
Q3 (49–54.9 U/L)	37/1,224(3.0)	1.56 (0.93–2.61)	1.48 (0.89–2.47)	56/1,243(4.5)	1.58 (1.04–2.41)	1.49 (0.98–2.27)
Q4 (55–61.9 U/L)	35/1,028(3.4)	1.75 (1.04–2.95)	1.49 (0.89–2.52)	48/1,041(4.6)	1.62 (1.05–2.49)	1.46 (0.95–2.26)
Q5 (≥62 U/L)	45/985(4.6)	2.35 (1.43–3.86)	1.91 (1.16–3.14)	63/1,003 (6.3)	2.21 (1.47–3.33)	1.93 (1.28–2.91)
**ALT**						
Q1 (0–10.0 U/L)	21/1,168(1.8)	1.00	1.00	49/1,196(4.1)	1.00	1.00
Q2 (10.1–12.9 U/L)	30/1,353(2.2)	1.23 (0.71–2.14)	1.11 (0.64–1.93)	38/1,361(2.8)	0.68 (0.45–1.03)	0.66 (0.43–1.00)
Q3 (13.0–15.9 U/L)	33/1,021(3.2)	1.79 (1.05–3.08)	1.60 (0.93–2.74)	41/1,029(4.0)	0.97 (0.65–1.46)	0.88 (0.59–1.33)
Q4 (16.0–24.9 U/L)	27/890(3.0)	1.68 (0.96–2.96)	1.45 (0.82–2.55)	57/920(6.2)	1.51 (1.04–2.19)	1.36 (0.93–1.97)
Q5 (≥25 U/L)	49/1,009(4.9)	2.70 (1.63–4.34)	2.10 (1.26–3.49)	59/1,019(5.8)	1.41 (0.97–2.05)	1.20 (0.79–1.68)
**AST**						
Q1 (0–14.9 U/L)	33/1,327 (2.5)	1.00	1.00	54/1,348(4.0)	1.00	1.00
Q2 (15.0–16.9 U/L)	40/1,334 (3.0)	1.20 (0.76–1.89)	1.33 (0.84–2.09)	52/1,346(3.8)	0.96 (0.66–1.40)	1.00 (0.68–1.46)
Q3 (17.0–18.9 U/L)	19/620 (3.1)	1.23 (0.71–2.15)	1.32 (0.75–2.30)	31/632(4.9)	1.22 (0.79–1.88)	1.26 (0.81–1.94)
Q4 (19.0–21.9 U/L)	38/1,273 (2.9)	1.20 (0.75–1.90)	1.31 (0.82–2.08)	61/1,296(4.7)	1.17 (0.82–1.68)	1.25 (0.87–1.79)
Q5 (≥ 22 U/L)	30/887 (3.4)	1.36 (0.83–2.21)	1.43 (0.88–2.35)	46/903(5.1)	1.27 (0.86–1.87)	1.28 (0.87–1.89)
**GGT**						
Q1 (0–9.9 U/L)	36/1,673(2.1)	1.00	1.00	45/1,682(2.7)	1.00	1.00
Q2 (10–12.9 U/L)	7/563 (1.2)	0.58 (0.26–1.29)	0.57 (0.25–1.27)	23/579(3.9)	1.48 (0.91–2.43)	1.42 (0.86–2.34)
Q3 (13–14.9 U/L)	36/1,337 (2.7)	1.25 (0.79–1.97)	1.12 (0.71–1.77)	57/1,358(4.2)	1.57 (1.07–2.30)	1.48 (1.01–2.17)
Q4 (15–19.9 U/L)	29/982 (2.95)	1.37 (0.85–2.22)	1.13 (0.69–1.84)	47/1,000(4.7)	1.75 (1.17–2.62)	1.58 (1.06–2.37)
Q5 (≥ 20 U/L)	53/886 (5.9)	2.27 (1.79–4.14)	1.96 (1.28–3.02)	72/906(7.9)	2.97 (2.06–4.27)	2.28 (1.57–3.32)

GH, Gestational hypertension; PE, Preeclampsia; BMI, body mass index; ALT, alanine aminotransferase; AST, aspartate aminotransferase; GGT, γ-glutamyl transferase; ALP, alkaline phosphatase.

* Based on percentile of concentrations. ALP, ALT, AST and GGT were divided into quintile groups (Q1: quintile 1, Q2: quintile 2, Q3: quintile 3, Q4: quintile 4, Q5: quintile 5) Continuous values of each liver enzyme test were divided into quintiles in multivariable log-binomial regression. We performed dummy variable alterations on quintile liver enzyme levels and used the 1st quintile as reference groups.

† The risk ratios were estimated by multivariable log-binomial regression, adjusting for age (years), preconception-BMI (normal: BMI <24 kg/m^2^ or overweight/obese: BMI ≥ 24 kg/m^2^), supplementation of folic acid before pregnancy (yes/no), smoking exposure during gestation (yes/no), whether gravidity > 1 (yes/no), gestational week and fasting glucose at the 1st antenatal visit. P-trend were estimated by multivariable log-binomial regression.

**Figure 3:**
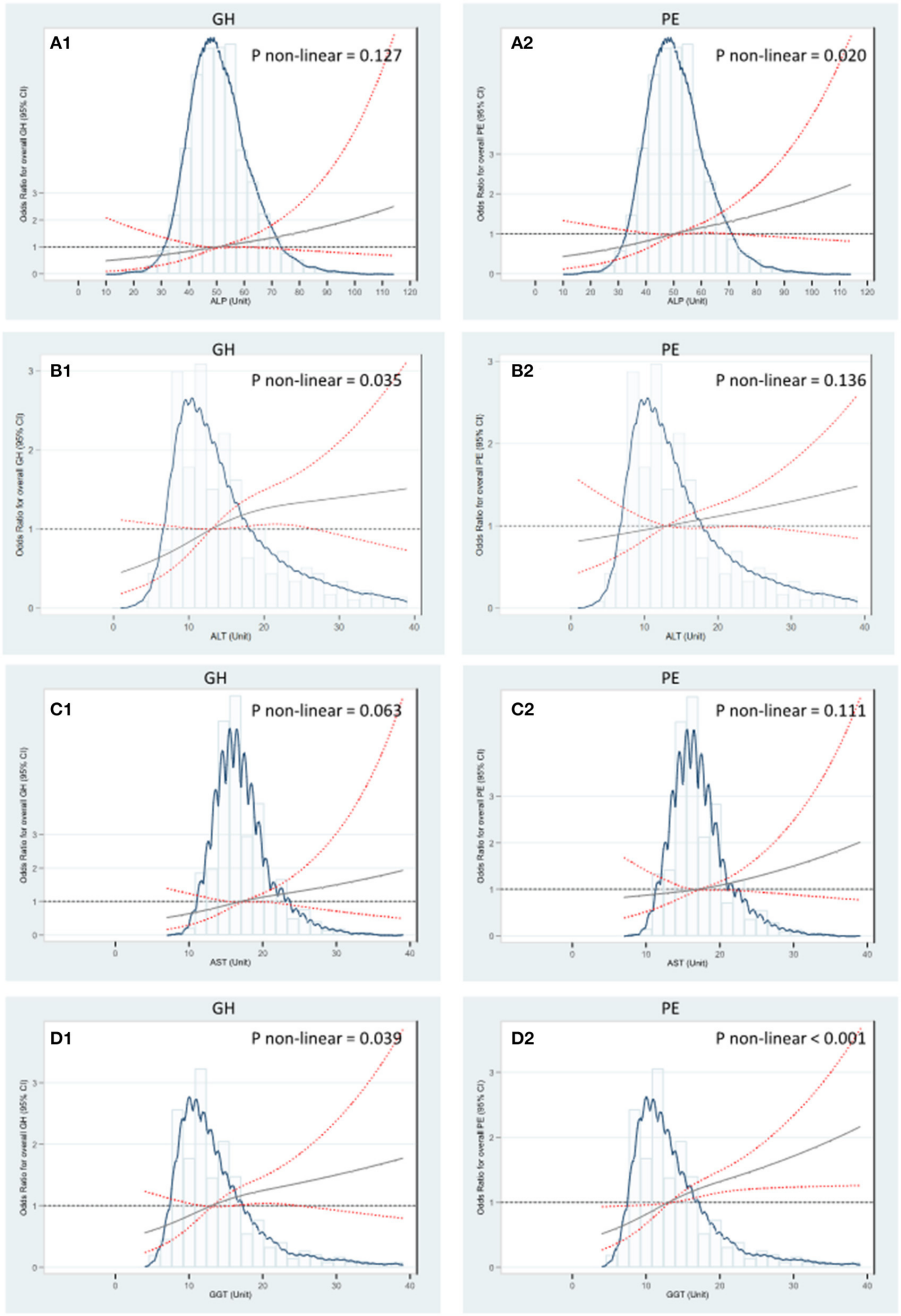
RCS regression analysis of liver enzyme levels with GH and PE. **(A)** The median of ALP 50 U/L was selected as the reference level to investigate associations of ALP with GH and PE. **(B)** The median of ALT 14 U/L was selected as the reference level to investigate associations of ALT with GH and PE. **(C)** The median of AST 17 U/L was selected as the reference level to investigate associations of AST with GH and PE. **(D)** The median of GGT 13 U/L was selected as the reference level to investigate associations of GGT with GH and PE. The Odds Ratios were estimated by multivariable logistic regression adjusting for age (years), preconception-BMI (normal: BMI <24 kg/m^2^ or overweight/obese: BMI ≥ 24 kg/m^2^), supplementation of folic acid (yes/no), smoking exposure during gestation (yes/no), whether gravidity > 1 (yes/no), gestational week and fasting glucose at the 1st antenatal visit. Non-linear tests were based on RCS models with three knots placed at the 10, 50, and 90th percentiles. The solid gray lines indicate estimated aORs and the red lines of dashes represent 95% CI. GH, Gestational hypertension; PE, Preeclampsia; GGT, γ-glutamyl transferase; ALT, alanine aminotransferase; ALP, alkaline phosphatase; AST, aspartate aminotransferase.

In addition, the RCS regression models showed that higher levels of ALT and GGT were associated with GH in a nonlinear manner, respectively (*P*_nonlinear_ = 0.035 and *P*_nonlinear_ = 0.039). The GH risk appeared linearly increased with liver enzyme levels while flattened off afterwards at 13 U/L for GGT and 14 U/L for ALT (Figures 3B1,D1). Similar nonlinear relationships of ALP and GGT with PE were also found (*P*_nonlinear_ = 0.020; *P*_nonlinear_ < 0.001, respectively) (Figures 3A2,D2). ALT did not show significant non-linear associations with GH or PE (Figures 3C1,C2). Compared with carrying none high-normal enzymes, the risk of GH significantly increased from 67 to 103% when exposed to one to four high-normal liver enzymes, while the increment of PE risk ranged from 52 to 46%, accordingly. The cumulative prevalence of GH and PE showed positive trends with given exposed one or up to four high-normal liver enzyme levels (all P_*trend*_ < 0.001) (Table 3). Of note, the cumulative PAF was 31.4% for GH and 23.2% for PE, respectively, indicating that nearly one fourth pregnant women with GH and PE could be attributed to affected one or more high-normal liver enzyme levels (Table 3).

**Table 3:** Risk ratios and population attributable risk of hypertensive disorders by numbers of affected high-normal liver enzymes in early gestation.

Number of affected high-normal liver enzymes^*^	**Prevalence rate**			**Unadjusted** ** RR (95% CI)**	**aRR (95% CI)**	**PAF^†^**
	**Yes, n (%)**	**No, n (%)**	**Total**			
**GH**						
0	54 (1.9)	2,800 (98.1)	2,947	1.00	1.00	-
1	47 (3.6)	1,263 (96.4)	1,380	1.89 (1.29–2.79)	1.67 (1.13–2.45)	19.3%
2	33 (4.3)	740 (94.7)	817	2.25 (1.47–3.45)	1.96 (1.25–3.04)	18.5%
3	18 (4.7)	363 (95.3)	411	2.49 (1.48–4.21)	2.03 (1.17–3.54)	12.7%
4	8 (6.5)	115 (93.5)	130	3.43 (1.67–7.06)	2.38 (1.07–5.30)	7.5%
Total	160 (2.9)	5,281 (97.1)	5,685	2.16 (1.57–2.99)	1.90 (1.36–2.66)	31.4%
**PE**						
0	90 (3.2)	2,800 (96.8)	2,947	1.00	1.00	-
1	70 (5.2)	1,263 (94.8)	1,380	1.64 (1.21–2.23)	1.52 (1.11–2.07)	14.5%
2	44 (5.6)	740 (94.4)	817	1.74 (1.23–2.48)	1.58 (1.10–2.26)	11.8%
3	30 (7.6)	363 (92.4)	411	2.37 (1.59–3.53)	1.92 (1.25–2.93)	11.5%
4	7(5.7)	115 (94.3)	130	1.78 (0.84–3.76)	1.46 (0.69–3.09)	2.2%
Total	244 (4.4)	5,281 (95.6)	5,685	1.78 (1.38–2.29)	1.60 (1.23–2.07)	23.2%

GH, gestational hypertension; PE, Preeclampsia.

* High-normal liver enzymes: liver enzyme levels equal or greater than the 5th quintiles of each liver enzyme, ALP > 62 U/L, ALT > 25 U/L, AST > 22 U/L and GGT > 20 U/L.

† PAF, population attributable fractions, was calculated from estimated proportions of pregnancies exposed to the 5th quintile ALP, ALT, AST and GGT respectively based on previous multivariable log-binomial regression models, which were ALP > 62 U/L, ALT > 25 U/L, AST > 22 U/L and GGT > 20 U/L.

^‡^The risk ratios were estimated by multivariable log-binomial regression, adjusting for age (years), preconception-BMI (normal: BMI <24 kg/m^2^ or overweight/obese: BMI ≥ 24 kg/m^2^), supplementation of folic acid before pregnancy (yes/no), smoking exposure during gestation (yes/no), whether gravidity > 1 (yes/no), gestational week and fasting glucose at the 1st antenatal visit.

### Sensitivity analysis

Including pregnant women with established liver diseases and clinically-abnormal liver enzyme levels, of note, the associations of elevated ALP and GGT levels with GH was similar (aRR, 1.19 [95% CI, 1.18–1.22] and 1.05 [1.01–1.09]). The associations with PE were similar for the four liver enzyme (1.15 [1.08–1.22] for ALP, 1.04 [1.01–1.07] for ALT, 1.04 [1.01–1.08] for AST, 1.11 [1.06–1.16] for GGT, respectively) (Supplementary Table S2).

## Discussion

Based on a prospective cohort of pregnant women with normal liver enzyme levels, we revealed that elevated ALP, ALT, and GGT levels in early pregnancy were associated with increased risk of GH. Of note, ALP and GGT remained positively associated with PE in a nonlinear fashion. One or up to four high-normal liver enzyme levels accounted for nearly one fourth pregnant women with GH and PE. Our study provided new evidence and advanced our understanding of the role of liver in HDP development and prevention. Prior observational studies reported clinically abnormal ALT and AST levels in early pregnancy as risk factors of HDP (5, 6, 17). A retrospective cohort study in East-Asian indicated that ALT > 95th percentage (30 U/L) in early pregnancy increased 3-fold risk of PE (7). A prospective cohort study in south China suggested that elevated GGT (16–68 U/L referred to 4–10 U/L) and ALP (53–193 U/L referred to 18–43 U/L) increased 2.6- and 2.1-times risk of HDP in early-pregnancy, respectively (8). A cross-sectional study in Bangladeshi non-pregnant population showed associations for continuous liver enzyme levels, that per unit increment in ALT, AST and GGT levels were associated with 4–7% increased risk of hypertension (18). To our knowledge, this is the first evidence that high-normal liver enzyme levels at early gestation are associated with subsequent increased risk of GH and PE among pregnant women with normal liver enzyme levels. The effect of liver enzymes in this study was similar but dampened compared to that in the previous findings. Our study suggested that the positive associations of liver enzyme levels with GH and PE still persisted in the normal range of liver function according to the criteria currently used in clinical practice.

### Liver enzymes

Biological mechanisms by which liver function confers increasing HDP risk are not well understood and our findings provide insight to this topic. Abundant evidences have shown strong associations of oxidative stress, dyslipidemia, and insulin intolerance with GH and PE (19–21). Liver enzymes may be involved in these pathways. In our study, GGT was the only liver enzyme that simultaneously showed significant associations with both GH and PE in a nonlinear fashion. According to previous studies, GGT was physiologically associated with regulation of insulin secretion and glucose tolerance (22), which were strong risk factors of HDP (23). Higher GGT level was also reported to be substantially related to increased level of ROS and catabolism of extracellular antioxidant glutathione in HDP development (24, 25). In our analysis, similar associations with GH were shown in GGT including participants with severe liver diseases. The RCS plot further presented steep curve until the GGT level arrived at 13 U/L and became flat afterwards. The biochemical mechanisms stated above might have reached maximum effects in the sub-range within the normal GGT level, although no previous studies have tested the assumption. Besides, both GGT and ALT may cause endothelial injury and vascular sclerosis through enhanced oxidative stress modification, which predispose great risk of HDP (26–28). It is consistent with our findings that elevated levels of GGT and ALT presented notable associations with GH. Of note, in our study, predominant ALP elevation was significantly associated with increased risk of PE. ALP played an important role in the oxidative stress dysregulation and endothelial dysfunction through purinergic signaling process in hypertension pathogenesis (29, 30). Furthermore, biomarkers for cholestatic liver disease (ALP and GGT) were more closely related to GH and PE than those indicating hepatocellular abnormalities (ALT and AST), which suggested that the occurrence of HDP may be related to predominant changes in bile duct function. This is supported by a study found that intrahepatic cholestasis of pregnancy increases the risk of PE (31). However, the exact mechanism of how biliary function affects the HDP pathogenesis remains unclear.

Elevation of liver enzyme levels is also a common clinical lab result of NAFLD (32). Driven by this, we think the association found in our study may partly due to excessive fat accumulation of liver in pregnant woman. NAFLD is assumed as one of the possible key etiologies in the development of HDP *via* mechanisms beyond overweight and obesity. The toxic fat-derived metabolites from ectopic fat accumulation initiate lipoprotein abnormalities and athrogenic dislipidemia, which instigate the pathophysiological changes in HDP development (33). The study by Sarkar et al. (34) supports our study findings that NAFLD during the perinatal period was an independent risk factor of HDP after adjusting for BMI. In our study, after adjustment on pre-pregnancy BMI, the strong associations persisted for liver enzyme levels increment with GH and PE. Unfortunately, neither image examination nor NAFLD diagnosis was available in our study. This assumption is expected to be further explored in future studies.

Our finding that nearly one fourth of pregnant women with GH and PE could be attributed to affected any one of the four high-normal liver enzyme levels are of great importance for clinical practice. Our findings support that it may be necessary to consider timely screening for high risk of HDP in pregnant women with high normal liver enzyme levels by liver function tests during early gestation (35). The high-normal liver enzyme levels in our study, defined as the 5th quintile levels (ALT: 25 vs. 40 U/L; AST: 22 vs. 40 U/L; ALP: 62 vs. 120 U/L; GGT: 20 vs. 40 U/L), will classify more pregnant women as high risk of HDP beyond current clinical reference values (13). P. Jamjute. et al suggested that the normal range of gestational liver enzyme levels in the 1st trimester should be lower than current reference cutoffs (10) (Supplementary Table S1). However, given differences across laboratories, further research is warranted to validate our findings.

### Strengths and limitations

The major strength was the prospective nature of our study. The positive dose-response associations suggested that the relations of liver function in early pregnancy with HDP may be causal. Besides, the liver enzyme levels in our study were within the normal range, which provided a new insight that hepatobiliary abnormalities may occur despite the normal biomarker levels, and the potential role of the insidious hepatobiliary dysfunction in early HDP pathogenesis. Our findings provided inspiration for future primary prevention of HDP in high-risk pregnant women. There were several limitations. Firstly, selection bias may exist because we only included subgroup of the large-scale SPCC cohort with measurements of liver enzymes in early pregnancy. Generalization of our findings to other regions requires further validation. Secondly, given the nature of observational study, the associations may be biased by unmeasured residual confounders, such as physical activities, kidney function (eGFR, creatinine, etc.), cardiovascular history (stroke, myocardial ischemia, etc.) and medication use (herbs and hepatic protectant). Third, although we first reported the significant association between high-normal liver enzyme levels and GH and PE respectively, the 5th quintile levels (ALT: 25 vs. 40 U/L, AST: 22 vs. 40 U/L, ALP: 62 vs. 120 U/L, and GGT: 20 vs. 40 U/L) we applied did not indicate the exact cutoffs due to the limited and nongeneralizable study population. Future studies are warranted to validate the best cutoff value for each enzyme in prediction of hypertensive disorders of pregnancy.

In conclusion, we provide the first evidence that elevated levels of ALP and GGT within the normal range in early pregnancy were associated with increased risk of GH and PE, respectively, followed by dose-response trends. Nearly one fourth of pregnant women with HDP could be attributed to any one of the four high-normal liver enzyme levels at early gestation. These results provide new insight into the role of hepatobiliary function in HDP pathogenesis. Pregnant women with high-normal liver enzyme levels in early pregnancy warrants attention for their subsequent risk of GH and PE.

## Data availability statement

The data generated and/or during current study are not publicly available, but are available from the corresponding author on reasonable request.

## Ethics statement

The studies involving human participants were reviewed and approved by Institutional Ethics Committee (Children's Hospital, Fudan University, No. 201649). The patients/participants provided their written informed consent to participate in this study.

## Author contributions

YZ and CS contributed to the statistical analysis, interpretation of data and manuscript drafting. XC, HC, YJ, YD, YW, DW, and ML the Shanghai PreConception Cohort Group were involved in recruitment, data and biosample collection. XC, YW, and YZ contributed to bio sample management and data management. WY, GH, HC, and WH made critical review. GH and WY obtained funding and contributed to the conception and design of the study had full access to all the data in the study and take responsibility for the integrity of the data and the accuracy of the data analysis. All authors contributed to results interpretation.

## Funding

This study is supported by the Natural Science Foundation of China (Grant No: 82070323), National key research and development program (Grant No: 2021YFC2701004), Research Project of Shanghai Municipal Health Commission (202140444), and CAMS Innovation Fund for Medical Sciences (2019-I2M-5-002).

## The Shanghai PreConception Cohort group

Guoying Huang, Weili Yan, Xiaojing Ma, Weifen Luo, Wei Sheng, Yi Zhang, Yuan Jiang, Ying Ye, Dingmei Wang, Xiaotian Chen, Yalan Dou, Yin Wang, Wei Sheng, Bing Jia, Mengru Li, Hongyan Chen, Xiangyuan Huang, Mi Ji, Yumei Liu, Qing Gu (s), Qing Gu (t), Xupeng Sun, Linmei Zhu, De'ai Hou, Peiyu Sun. (Children's Hospital of Fudan University, Shanghai, China), Hongbing Wang, Li Meng, Lin Zhang (Jingan Maternal and Child Health Center), Zifen Dai, Li Feng (Shanghai First Maternity and Infant health Hospital), Shufang Chen, Zhenhua Tang, Jiahao Wu (International Peace Maternal and Child Health Hospital), Shuhua Wang, Dan Li, Hui Wang (Xuhui Maternal and Child Health Center), Yu Ke, Weiping Cao, Baoren Zhang, Hong Huang (Shanghai Pudong New Area Health Care Hospital for Women & Children), Nailing Wang, Min Jiang, Jie Chen, Qiumin Xia (Shanghai Punan Hospital of Pudong New District), Hui Xu, Guoying Lao (Changning Maternity and Infant Health Hospital), HongMei Jin, Wenjuan Xie, Pin Yi (Qingpu Hospital, Zhongshan Hospital), Weiming Gong, JianXin Xu, Yingying Qian (Shanghai Qingpu Maternal and Child Health Center), Mingjie Luo, Jingwei Xia, Dongmei Chen, Zhenyu Tang (Shanghai Huangpu Maternal and Child Health Center), Xuejing Zhu, Qing Liu, Huiling Yang (Shanghai Huangpu Maternal and Child Health Hospital), Xiaotian Li, Zhiyong Wu, Chuanmin Yin, Shan Shi [Obstetrics and Gynecology Hospital of Fudan University (Shanghai Red House Obstetrics and Gynecology Hospital)], Yanquan Zhang, Mingyi Yang (Wujing Hospital, Minhang District, Shanghai), Xiaohua Zhang, Lei Zhang, Lin Guan (Shanghai Minhang District Maternal and Child Health Care Hospital), Jinyu Xu, Honglin Wang, Fang Shen (The Fifth People's Hospital of Shanghai, Fudan University), Wenying Li, Xiaojing Teng, Jinling Zhao (Shanghai Minhang TCM Hospital), Cuili Zhu, Lan Wang, Hongwei Chen (Shanghai Songjiang District Central Hospital), Xiaoming Yuan, Meihua Zhang, Yaqiong Jin (Sijing Hospital, Songjiang District, Shanghai), Qing Yang, Hong Zhu, Min Feng (Songjiang Maternal and Child Health Center), Ying Wang, Yan Wu, Hong Tang (Songjiang Maternal and Child Health Hospital), Sa Guo (Tongji Hospital of Tongji University), Hongling Du (Shanghai Putuo District People's Hospital), Yuhuan Liu, Zhanyue Yi, Renhua Shi (Changhai Hospital, Second Military Medical University, Shanghai), Yu Gu, Qinfen Su, Yingying Lv (Shanghai Zhabei District Central Hospital), Yun Sun, Qiongpei Gu (Yangpu District Family Planning Service Center), Xixia Pang, Qingwu Zhang (Kong Jiang Hospital of Yangpu District, Shanghai), Songxiao Bai, Baoqiao Qi (Shanghai East City Hospital), Meijuan Lu, Jianwei Hu, Xia Han (Women and Children in Kunshan, Jiangsu).

## Conflict of interest

The authors declare that the research was conducted in the absence of any commercial or financial relationships that could be construed as a potential conflict of interest.

## Publisher's note

All claims expressed in this article are solely those of the authors and do not necessarily represent those of their affiliated organizations, or those of the publisher, the editors and the reviewers. Any product that may be evaluated in this article, or claim that may be made by its manufacturer, is not guaranteed or endorsed by the publisher.
