# Supplementary material for: High-normal liver enzyme levels in early pregnancy predispose the risk of gestational hypertension and preeclampsia: A prospective cohort study
Source: Front Cardiovasc Med. 2022 Sep 12;9:963957. doi: 10.3389/fcvm.2022.963957 (PMC9510982; doi:10.3389/fcvm.2022.963957)
Supplement: Supplementary file 1 [file Data_Sheet_1.docx]

Supplementary files for

**High-normal Liver Enzyme Levels in Early Pregnancy Predispose the Risk of Gestational Hypertension and Preeclampsia: A Prospective Cohort Study**

Yi Zhang^1,2†^,MPH ,PhD; Chen Sheng^1,2†^,MD; Dingmei Wang^1,2^,PhD; Xiaotian Chen^1,2^,MS; Yuan Jiang^1,2^,MS; Yalan Dou^1,2^,PhD; Yin Wang^1,2^,PhD; Mengru Li^1,2^,MD; Hongyan Chen^1,2^,PhD; Wennan He^1,2^,MS; Weili Yan^1,2,3*^,PhD; Guoying Huang^1,2,3*^,MD,PhD; On behalf of the SPCC group

**Affiliations:**

1. Children's Hospital of Fudan University, Shanghai, China.
2. Shanghai Key Lab of Birth Defect, Shanghai, China.
3. Research Unit of Early Intervention of Genetically Related Childhood Cardiovascular Diseases(2018RU002), Chinese Academy of Medical Sciences.

^†^ These authors have contributed equally to this work and share first authorship

* Equal corresponding authors

***Corresponding authors:**

**Guoying Huang, MD, PhD, gyhuang@shmu.edu.cn**

Pediatric Heart Center, Children’s Hospital of Fudan University, National Children’s Medical Center, Shanghai, China & Shanghai Key Laboratory of Birth Defects, Shanghai, China;

Address: 399 Wan Yuan Road, Shanghai 201102, People’s Republic of China

Tel: 86-21-64931928

Fax: 86-21-64931002

**Weili Yan PhD, yanwl@fudan.edu.cn**

Department of Clinical Epidemiology & Clinical Trial Unit, Children’s Hospital of Fudan University, National Children’s Medical Center, Shanghai, China & Shanghai Key Laboratory of Birth Defects, Shanghai, China;

Address: 399 Wan Yuan Road, Shanghai 201102, People’s Republic of China

Tel: +86-021-64931215

Fax: +86-021-64931215

**
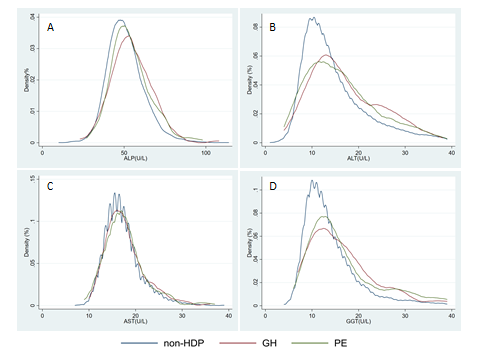
**

**Supplementary Figure 1** Distribution of the four liver enzyme levels in early pregnancy among non-HDP, GH and PE. HDP, Hypertension disorder in pregnancy; GH, Gestational hypertension; PE, Preeclampsia; ALP, alkaline phosphatase(A); ALT, alanine aminotransferase(B); AST, aspartate aminotransferase(C); GGT, γ-glutamyl transferase(D)

**Supplementary Table 1** The 80^th^ percentile levels of liver enzymes in the current study and the references published

| **Liver enzymes** | **80^th^ percentage level in current study** | **American Board of Internal Medicine Laboratory Test Reference Ranges [13]** | **The** [**upper**](javascript:;) [**limit**](javascript:;) **of pregnancy liver enzyme normal range [4]** |
| --- | --- | --- | --- |
| ALP (IU/L) | 62 | 120 | 100 |
| ALT (IU/L) | 25 | 40 | 32 |
| AST (IU/L) | 22 | 40 | 28 |
| GGT (IU/L) | 20 | 40 | 37 |

ALP, alkaline phosphatase; ALT, alanine aminotransferase; AST, aspartate aminotransferase; GGT, γ-glutamyl transferase.

**Supplementary Table 2** Associations of liver enzyme levels in early pregnancy with GH and PE in the study population including pregnant women with established liver diseases and clinically-abnormal liver enzyme levels

| **Liver enzymes**  **(per 1 SD)** | **Adjusted Risk Ratio*** | **P value** |
| --- | --- | --- |
| **GH** |  |  |
| ALP | 1.19 (1.18, 1.22) | <0.001 |
| ALT | 1.02 (0.98 ,1.06) | 0.253 |
| AST | 1.01 (0.96 ,1.06) | 0.598 |
| GGT | 1.05 (1.01 ,1.09) | 0.019 |
|  |  |  |
| **PE** |  |  |
| ALP | 1.15 (1.08, 1.22) | <0.001 |
| ALT | 1.04 (1.01 ,1.07) | 0.009 |
| AST | 1.04 (1.01 ,1.08) | 0.020 |
| GGT | 1.11 (1.06 ,1.16) | <0.001 |

*The risk ratios and 95%CIs were estimated by multivariable log-binomial regression adjusting for age (years), preconception-BMI (normal: BMI<24 kg/m^2^ or overweight/obese: BMI≥24 kg/m^2^), supplementation of folic acid before pregnancy (yes/no), smoking exposure during gestation (yes/no), whether gravidity>1 (yes/no), gestational week and fasting glucose at the 1^st^ antenatal visit. GH, Gestational hypertension; PE, Preeclampsia; ALP, alkaline phosphatase; ALT, alanine aminotransferase; AST, aspartate aminotransferase; GGT, γ-glutamyl transferase.
